# Phylogenetic Analysis of K^+^ Transporters in Bryophytes, Lycophytes, and Flowering Plants Indicates a Specialization of Vascular Plants

**DOI:** 10.3389/fpls.2012.00167

**Published:** 2012-08-02

**Authors:** Judith Lucia Gomez-Porras, Diego Mauricio Riaño-Pachón, Begoña Benito, Rosario Haro, Kamil Sklodowski, Alonso Rodríguez-Navarro, Ingo Dreyer

**Affiliations:** ^1^Centro de Biotecnología y Genómica de Plantas, Universidad Politécnica de MadridMadrid, Spain; ^2^Grupo de Biología Computacional y Evolutiva, Departamento de Ciencias Biológicas, Universidad de los AndesBogotá D.C., Colombia; ^3^Institut für Biochemie und Biologie, Universität PotsdamPotsdam, Germany; ^4^Max-Planck-Institute of Molecular Plant PhysiologyPotsdam-Golm, Germany

**Keywords:** potassium, transport, channel, voltage-dependent, voltage-independent, high-affinity, *Selaginella*

## Abstract

As heritage from early evolution, potassium (K^+^) is absolutely necessary for all living cells. It plays significant roles as stabilizer in metabolism and is important for enzyme activation, stabilization of protein synthesis, and neutralization of negative charges on cellular molecules as proteins and nucleic acids. Land plants even enlarged this spectrum of K^+^ utilization after having gone ashore, despite the fact that K^+^ is far less available in their new oligotrophic habitats than in sea water. Inevitably, plant cells had to improve and to develop unique transport systems for K^+^ accumulation and distribution. In the past two decades a manifold of K^+^ transporters from flowering plants has been identified at the molecular level. The recently published genome of the fern ally *Selaginella moellendorffii* now helps in providing a better understanding on the molecular changes involved in the colonization of land and the development of the vasculature and the seeds. In this article we present an inventory of K^+^ transporters of this lycophyte and pigeonhole them together with their relatives from the moss *Physcomitrella patens*, the monocotyledon *Oryza sativa*, and two dicotyledonous species, the herbaceous plant *Arabidopsis thaliana*, and the tree *Populus trichocarpa*. Interestingly, the transition of green plants from an aqueous to a dry environment coincides with a dramatic reduction in the diversity of voltage-gated potassium channels followed by a diversification on the basis of one surviving K^+^ channel class. The first appearance of K^+^ release (K_out_) channels in *S. moellendorffii* that were shown in *Arabidopsis* to be involved in xylem loading and guard cell closure coincides with the specialization of vascular plants and may indicate an important adaptive step.

## Introduction

The absolute requirement for K^+^in all living cells was already fixed from the cradle of evolution in the sea. Among all the cations that were present in the marine environment K^+^ was utilized by cells as the major cation for essential functions as maintaining electroneutrality and osmotic equilibrium. Further evolutionary steps in the cellular K^+^-rich environment then employed K^+^ as regulator of protein activities being essential for several biochemical processes. The interactions of potassium with these proteins depend on the unique electrochemical properties of K^+^ ions, i.e., the topology of their electrical charge-density. These features cannot or only incompletely be mimicked by Na^+^ or by any other cation because they all differ from K^+^ in their electron shell configuration and consequently also in the arrangement of the surrounding hydration shell. K^+^ thus became indispensably necessary for living cells; a dependency also inherited to Embryophyta, where K^+^ can contribute up to 10% of the dry mass (Leigh and Wyn Jones, 1984). Terrestrial plants even developed new functions for K^+^ such as turgor-driven processes like stomatal movement, phototropism, gravitropism, and cell elongation (Ashley et al., 2006; Rodriguez-Navarro and Rubio, 2006; Amtmann and Armengaud, 2009; Amtmann and Blatt, 2009; Maathuis, 2009; Szczerba et al., 2009). Embryophyta need to survive in oligotrophic environments where K^+^ is present at much lower concentrations than in sea water; the potassium concentration in normal soil solution (10–100 μM) is considerably variable and about three to four orders of magnitude lower than in the plant. Therefore, not only for potassium homeostasis (the maintenance of a dynamic equilibrium in the cellular K^+^ concentration) but also for K^+^ uptake from the environment and its distribution throughout the organism, plants have to invest energy and need a set of specialized transporter proteins.

Pioneering work by Epstein et al. (1963) proposed that K^+^ uptake from soil into plant cells is mediated by two mechanisms that take advantage of the electrical gradient and/or the proton motive force established by H^+^-ATPases. One was characterized as a high-affinity system (mechanism I), showing apparent affinities in the range of ∼20 μM, that can transport also Na^+^ when K^+^ is not present. The other one (mechanism II) showed a much lower affinity and provided an increasing contribution from >200 μM to mM external K^+^ concentrations. During the last two decades a variety of potassium-permeable transmembrane transport systems – potentially underlying these two components – were identified at the molecular level. They were classified into five major gene families (Maser et al., 2001, 2002b; Véry and Sentenac, 2002, 2003; Lebaudy et al., 2007): (i) voltage-gated K^+^ channels, (ii) non-voltage-gated (tandem-pore) K^+^(TPK) channels, (iii) high-affinity K^+^ transporters of the HAK type, (iv) high-affinity K^+^transporters of the HKT type, and (v) cation-proton antiporters (CPAs). K^+^ channels likely underlie the experimentally observed low affinity component in plants, whereas HAK transporters contribute to the high-affinity K^+^ uptake component. HKT transporters are responsible for a K^+^-dependent Na^+^ component (Rodriguez-Navarro and Rubio, 2006). However, this is not a generalized strict separation. Channels contribute to the high-affinity K^+^ uptake component and transporters might contribute under certain conditions also to low affinity transport.

Here we took advantage from the recently published genome of *Selaginella moellendorffii* (Banks et al., 2011) and prepared an inventory of K^+^ transporters in this lycophyte. We focused especially on transporters of the HAK and HKT type and on K^+^ channels. For information on other potentially K^+^-permeable transporters such as KEA or CHX belonging to the class of monovalent CPAs we refer to a recent excellent review especially dedicated to these proteins (Chanroj et al., 2012). We are comparing the results from *S. moellendorffii* with those from the moss *Physcomitrella patens*, the monocotyledon *Oryza sativa*, as well as with those from two dicotyledonous species, the Brassicaceae *Arabidopsis thaliana*, and the tree *Populus trichocarpa*. Voltage-gated K^+^ channels are also compared with those from Chlorophyta.

## Results and Discussion

### Potassium transporters of the HAK type

The high-affinity K^+^ (HAK) transporter gene family – also called KT or KUP transporter family – is an ancient large family with members in Bacteria, Archaea, Fungi, Amoebozoa, and probably also in some species of Animalia (Grabov, 2007; Benito et al., 2011). Initially HAK genes have been deduced from plants by their similarity to K^+^ uptake permeases (KUP) from *E. coli* (Schleyer and Bakker, 1993) and high-affinity K^+^ transporters (HAK) from fungi (Banuelos et al., 1995; Quintero and Blatt, 1997; Santa-Maria et al., 1997; Fu and Luan, 1998; Kim et al., 1998). Several members of this family were shown to function as K^+^ uptake transporters in plants especially when the external potassium concentration was in the low μM range (Gierth et al., 2005; Aleman et al., 2011) indicating that HAK transporters are involved in high-affinity K^+^ uptake. Interestingly, all plant genomes analyzed so far contain genes encoding HAK transporters, while in Bacteria, Archaea, and Fungi they were found only in a subset of species (Grabov, 2007; Benito et al., 2011). The HAK family is the largest family of potential K^+^ transporters in plants and members of this family are expressed in nearly all tested plant tissues suggesting that HAK transporters have a general function in K^+^ supply (Banuelos et al., 2002).

**Figure 1:**
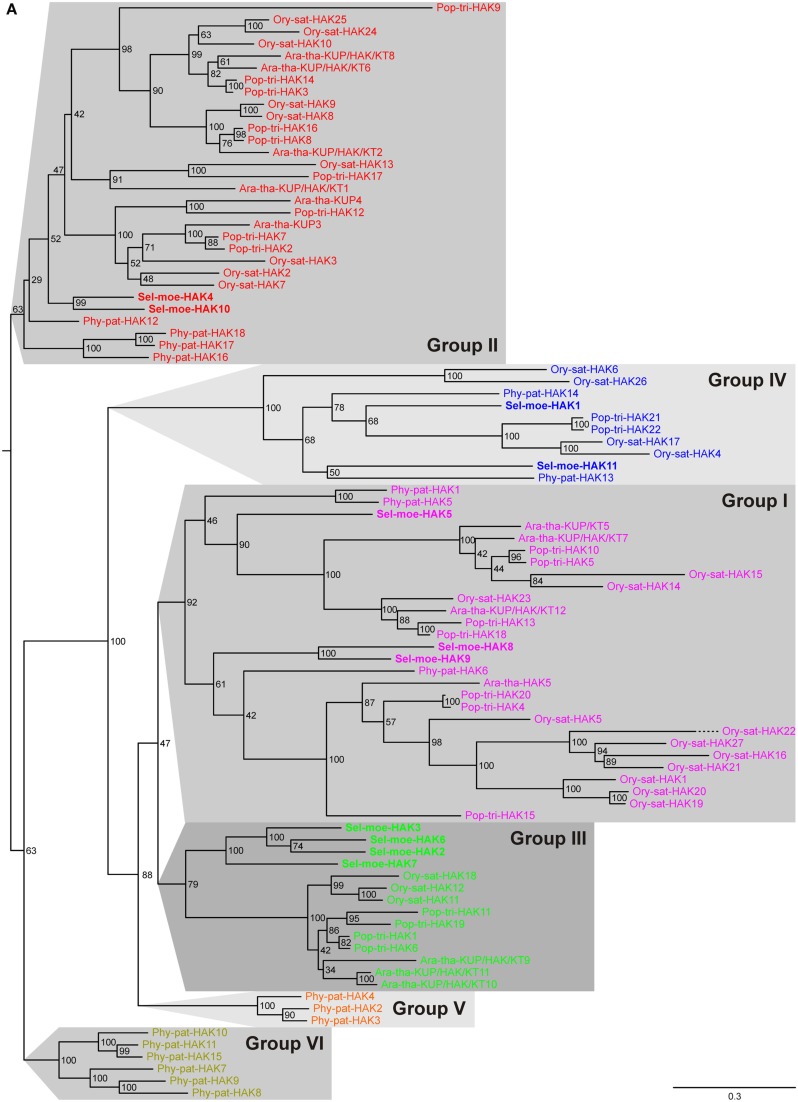

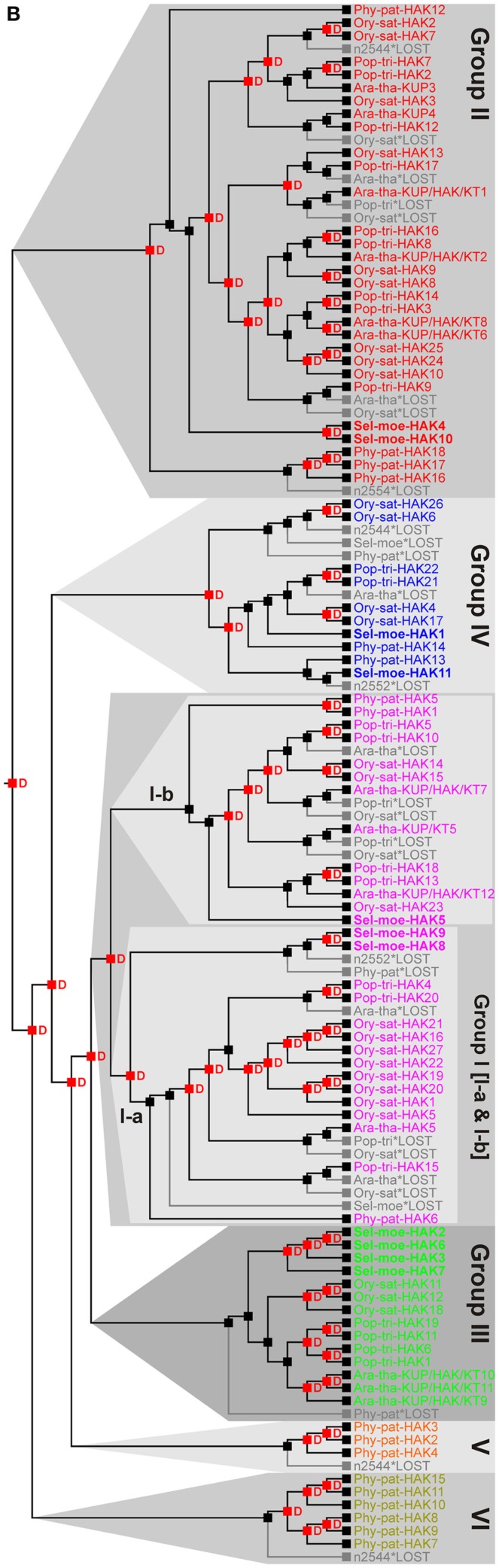
**Evolutionary relationships among HAK transporters in land plants**. **(A)** There are six clearly distinguished clades of HAK transporters in extant land plants, i.e., Groups I, II, III, IV, V, and VI. Each group represents an independent group of orthologs. Groups V and VI are *P. patens*-specific gene family amplifications. **Evolutionary relationships among HAK transporters in land plants**. **(B)** Reconciliation analysis of HAK transporters. The last recent common ancestor of all embryophytes had two HAK transporters. One of these diverged into current Group II, whereas the other was duplicated at least three times before the origin of tracheophytes. Two of these duplications got lost in the lineage leading to tracheophytes forming *P. patens*-specific groups. Red “D”s at branching points indicate predicted gene duplications. Gray branches indicate gene losses.

To date, the topology of HAK transporters has neither been determined experimentally nor by *in silico* predictions; nevertheless, hydropathy profiles of these proteins suggest about 12 putative transmembrane segments and a long hydrophilic COOH-terminal region. In genome-wide screenings, HAK transporter proteins can be pinpointed by the presence of several conserved consensus motifs (see Materials and Methods). Our screenings identified 13 HAKs in *Arabidopsis*, 27 in rice, 22 in poplar, 18 in *P. patens*, and 11 in *S. moellendorffii* (Table 1; see Yang et al., 2009, for comparison). It is likely that the genome of *P. trichocarpa* contains more genes coding for HAK transporters, because especially in the screening of poplar we discarded partial sequences resulting from pre-mature gene annotation. Phylogenetic analyses allowed subdividing them into six independent groups (Figure 1A; see Rubio et al., 2000, for initial grouping-into Groups I–IV) and revealed that the last recent common ancestor of all embryophytes had two HAK transporters (Figure 1B). One of these diverged into current Group II, whereas the other was duplicated at least three times before the origin of tracheophytes. Two early duplication events got lost in the lineage leading to tracheophytes and led to *P. patens*-specific gene family amplifications (Groups V and VI). HAK transporters in *S. moellendorffii* spread over the other clades (Groups I–IV).

**Table 1:** **Transporters of the HAK type presented in this study**.

Species	Locus	Name
*A. thaliana*	AT2G30070	Ara-tha-KUP/HAK/KT1
	AT2G40540	Ara-tha-KUP/HAK/KT2
	AT3G02050	Ara-tha-KUP3
	AT4G23640	Ara-tha-KUP4
	AT4G33530	Ara-tha-KUP/KT5
	AT4G13420	Ara-tha-HAK5
	AT1G70300	Ara-tha-KUP/HAK/KT6
	AT5G09400	Ara-tha-KUP/HAK/KT7
	AT5G14880	Ara-tha-KUP/HAK/KT8
	AT4G19960	Ara-tha-KUP/HAK/KT9
	AT1G31120	Ara-tha-KUP/HAK/KT10
	AT2G35060	Ara-tha-KUP/HAK/KT11
	AT1G60160	Ara-tha-KUP/HAK/KT12
*O. sativa*	LOC_Os04g32920	Ory-sat-HAK1
	LOC_Os01g70940	Ory-sat-HAK2
	LOC_Os01g27170	Ory-sat-HAK3
	LOC_Os08g36340	Ory-sat-HAK4
	LOC_Os01g70490	Ory-sat-HAK5
	LOC_Os01g70660	Ory-sat-HAK6
	LOC_Os07g47350	Ory-sat-HAK7
	LOC_Os03g21890	Ory-sat-HAK8
	LOC_Os07g48130	Ory-sat-HAK9
	LOC_Os06g42030	Ory-sat-HAK10
	LOC_Os04g52390	Ory-sat-HAK11
	LOC_Os08g10550	Ory-sat-HAK12
	LOC_Os06g45940	Ory-sat-HAK13
	LOC_Os07g32530	Ory-sat-HAK14
	LOC_Os04g52120	Ory-sat-HAK15
	LOC_Os03g37840	Ory-sat-HAK16
	LOC_Os09g27580	Ory-sat-HAK17
	LOC_Os09g38960	Ory-sat-HAK18
	LOC_Os02g31910	Ory-sat-HAK19
	LOC_Os02g31940	Ory-sat-HAK20
	LOC_Os03g37930	Ory-sat-HAK21
	LOC_Os07g01214	Ory-sat-HAK22
	LOC_Os09g21000	Ory-sat-HAK23
	LOC_Os06g15910	Ory-sat-HAK24
	LOC_Os02g49760	Ory-sat-HAK25
	LOC_Os08g39950	Ory-sat-HAK26
	LOC_Os03g37830	Ory-sat-HAK27
*P. trichocarpa*	POPTR_0001s00590	Pop-tri-HAK1
	POPTR_0002s23850	Pop-tri-HAK2
	POPTR_0010s10440	Pop-tri-HAK3
	POPTR_0001s03680	Pop-tri-HAK4
	POPTR_0003s01820	Pop-tri-HAK5
	POPTR_0003s10910	Pop-tri-HAK6
	POPTR_0014s14180	Pop-tri-HAK7
	POPTR_0013s08110	Pop-tri-HAK8
	POPTR_0015s05040	Pop-tri-HAK9
	POPTR_0001s21310	Pop-tri-HAK10
	POPTR_0003s10920	Pop-tri-HAK11
	POPTR_0003s13370	Pop-tri-HAK12
	POPTR_0008s14660	Pop-tri-HAK13
	POPTR_0008s14670	Pop-tri-HAK14
	POPTR_0014s12700	Pop-tri-HAK15
	POPTR_0019s08430	Pop-tri-HAK16
	POPTR_0003s14800	Pop-tri-HAK17
	POPTR_0010s10450	Pop-tri-HAK18
	POPTR_0001s00580	Pop-tri-HAK19
	POPTR_0001s12790	Pop-tri-HAK20
	POPTR_0008s14040	Pop-tri-HAK21
	POPTR_0010s11100	Pop-tri-HAK22
*P. patens*	Pp1s6_102V6	Phy-pat-HAK1
	Pp1s118_70V6	Phy-pat-HAK2
	Pp1s143_101V6	Phy-pat-HAK3
	Pp1s96_141V6	Phy-pat-HAK4
	Pp1s74_90V6	Phy-pat-HAK5
	Pp1s29_214V6	Phy-pat-HAK6
	Pp1s16_292V6	Phy-pat-HAK7
	Pp1s244_62V6	Phy-pat-HAK8
	Pp1s19_61V6	Phy-pat-HAK9
	Pp1s251_25V6	Phy-pat-HAK10
	Pp1s33_316V6	Phy-pat-HAK11
	Pp1s165_138V6	Phy-pat-HAK12
	Pp1s134_179V6	Phy-pat-HAK13
	Pp1s166_51V6	Phy-pat-HAK14
	Pp1s488_12V6	Phy-pat-HAK15
	Pp1s25_346V6	Phy-pat-HAK16
	Pp1s91_133V6	Phy-pat-HAK17
	Pp1s201_129V6	Phy-pat-HAK18
*S. moellendorffii*	PACid_15405883	Sel-moe-HAK1
	PACid_15408107	Sel-moe-HAK2
	PACid_15409215	Sel-moe-HAK3
	PACid_15411376	Sel-moe-HAK4
	PACid_15411378	Sel-moe-HAK5
	PACid_15413823	Sel-moe-HAK6
	PACid_15413143	Sel-moe-HAK7
	PACid_15422615	Sel-moe-HAK8
	PACid_15403105	Sel-moe-HAK9
	PACid_15404811	Sel-moe-HAK10
	PACid_15410020	Sel-moe-HAK11

Functional information on HAK transporters is unfortunately still scarce. Most data are available for HAK transporters belonging to Group I. Ara-tha-HAK5, Ory-sat-HAK1, Ory-sat-HAK5, and Phy-pat-HAK1 were characterized as high-affinity K^+^ transporters (Rubio et al., 2000; Banuelos et al., 2002; Gierth et al., 2005; Garciadeblas et al., 2007; Qi et al., 2008; Horie et al., 2011b). It might thus be an educative guess to propose high-affinity K^+^-uptake properties also for the *S. moellendorffii* orthologs in the same clade, i.e., Sel-moe-HAK5, Sel-moe-HAK8, and Sel-moe-HAK9.

HAK transporters may not only mediate transport across the plasma membrane. Transient expression of the Ory-sat-HAK10::GFP fusion protein in living onion epidermal cells targeted this protein (from Group II) to the tonoplast (Banuelos et al., 2002); and Ara-tha-KUP/HAK/KT12 (from Group I) was found in the chloroplast proteome (Kleffmann et al., 2004; Peltier et al., 2004). Additionally, HAK transporters may not exclusively transport K^+^. Phy-pat-HAK1 and Ara-tha-HAK5, for instance, were reported to be permeable also for Cs^+^ (Garciadeblas et al., 2007; Qi et al., 2008); and Phy-pat-HAK13 belonging to Group IV was recently characterized as a high-affinity Na^+^ uptake transporter (Benito et al., 2012). We therefore propose for the closely related Sel-moe-HAK1 and Sel-moe-HAK11 from *S. moellendorffii* similar sodium-transport features. This phylogenetic divergence may indicate a – so far underexplored – diversity of HAK transporters in fine-tuned function of K^+^ uptake and re-distribution, cellular expression, and/or sub-cellular targeting.

### Potassium transporters of the HKT type

**Figure 2:**
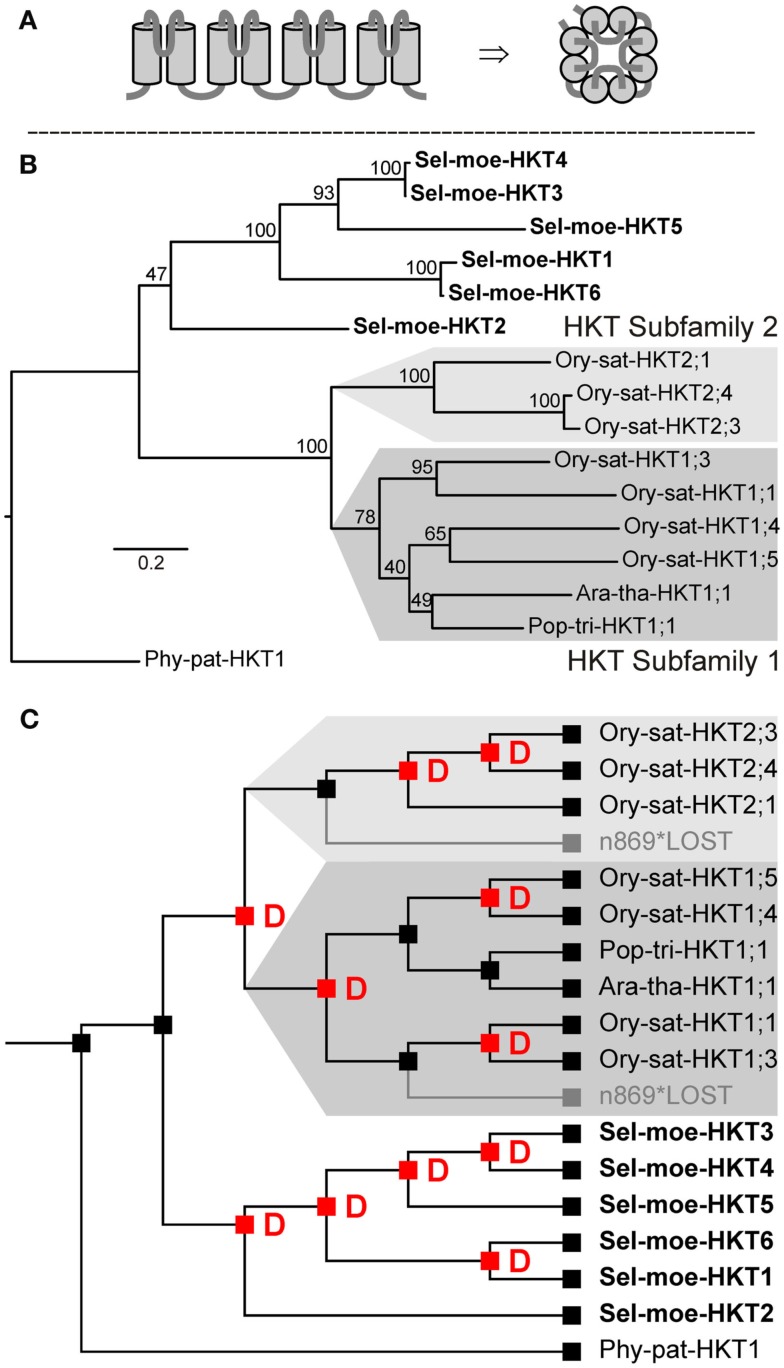
**Transporters of the HKT type in land plants**. **(A)** Predicted structure with a fourfold TM-P-TM motif in side view (left) and assembled as functional transporter in top view (right). **(B)** Evolutionary relationships among HKTs in land plants. This family is represented by a single group of orthologs that includes all considered extant land plants. **(C)** Reconciliation analysis of transporters of the HKT type. The last recent common ancestor of embryophytes had a single HKT-coding gene. Successively this family has undergone independent gene amplifications in different lineages, i.e., lycophytes and angiosperms. Red “D”s at branching points indicate predicted gene duplications. Gray branches indicate gene losses.

HKTs in plants belong to a family of monovalent cation transporters comprising also the fungal TRKs (K^+^ transporters) and bacterial KtrABs (Na^+^-dependent K^+^ transporter), for instance (Corratge-Faillie et al., 2010). Proteins of this family share a common structure of four TM-P-TM motifs (every two transmembrane α-helices are connected by ∼30 aa-long pore-forming P segments), which might have evolved from an ancestor related to the bacterial KscA K^+^ channel of *Streptomyces lividans* (Durell and Guy, 1999; Figure 2A). The plant HKT family comprises transporters that mediate Na^+^ uptake in roots or in other plant organs. They accumulate Na^+^ from the soil and recirculate it throughout the plant. There are two types of plant HKT transporters that can be distinguished by the amino acid sequence of the selectivity filter (the narrowest part of the permeation pathways that selects one ion species over others) of the first TM-P-TM motif: (i) S-S-M and (ii) [T,S,I]-G-L. Two HKTs from *O. sativa* and *A. thaliana* belonging to the first type have been well characterized *in planta* as Na^+^ uptake transporters (Uozumi et al., 2000; Rus et al., 2001; Maser et al., 2002a,c; Berthomieu et al., 2003; Garciadeblas et al., 2003; Sunarpi et al., 2005; Horie et al., 2007; Xue et al., 2011). The function of the second type has not been studied in plants. Nonetheless, two members of this group, from barley and wheat, mediate K^+^ or Na^+^uniport or Na^+^-K^+^symport – depending on the protein expression level – when heterologously expressed in yeast cells (Haro et al., 2005; Banuelos et al., 2008). Functional expression of this type of transporters in *Xenopus* oocytes produced similar results with slight variations regarding K^+^ versus Na^+^ permeability, symport activity, and permeability to divalent cations (Rubio et al., 1995; Gassman et al., 1996; Jabnoune et al., 2009; Lan et al., 2010; Horie et al., 2011a; Oomen et al., 2012).

In genome-wide screenings, proteins of the HKT type can be pinpointed by the presence of several conserved consensus motifs (see Materials and Methods). Our screenings identified one HKT-coding gene in *Arabidopsis*, seven in rice, one in poplar, one in *P. patens*, and six in *S. moellendorffii* (Table 2). Phylogenetic analyses grouped all of them into a single group of orthologs (Figure 2B) indicating that the most recent common ancestor of all embryophytes comprised a single protein of the HKT type. *P. patens* has a single extant representative (Phy-pat-HKT1), whereas in tracheophytes several duplication events occurred in different lineages (Figure 2C). The obviously independent multiplication of HKT-coding genes in rice and *S. moellendorffii* may be correlated with the affinity of these vascular plants to moisture environments. Probably, a larger variety of Na^+^/K^+^ transporters provides some advantage for better adaptation. Initially, the HKT family has been partitioned into the two subfamilies one and two, and transporter nomenclature was adjusted accordingly of the type “*species* HKT *subfamily*; *No*” (Platten et al., 2006). Subfamily one gathers transporters with the S-S-M signature in the selectivity filter of the first TM-P-TM motif. Our analysis now reveals that this subfamily division emerged in land plants only after the separation of Lycopodiophyta. Thus, the proposed unified nomenclature cannot be applied to all plant HKT genes. The rules fail, for instance, for HKTs from *S. moellendorffii* and *P. patens* (see also Haro et al., 2010).

**Table 2:** **Transporters of the HKT type presented in this study**.

Species	Locus	Name
*A. thaliana*	AT4G10310	Ara-tha-HKT1;1
*O. sativa*	LOC_Os04g51820	Ory-sat-HKT1;1
	LOC_Os02g07830	Ory-sat-HKT1;3
	LOC_Os04g51830	Ory-sat-HKT1;4
	LOC_Os01g20160	Ory-sat-HKT1;5
	LOC_Os06g48810	Ory-sat-HKT2;1
	LOC_Os01g34850	Ory-sat-HKT2;3
	LOC_Os06g48800	Ory-sat-HKT2;4
*P. trichocarpa*	POPTR_0018s13210	Pop-tri-HKT1;1
*P. patens*	Pp1s63_164V6	Phy-pat-HKT1
*S. moellendorffii*	PACid_15414191	Sel-moe-HKT1
	PACid_15414777	Sel-moe-HKT2
	PACid_15422070	Sel-moe-HKT3
	PACid_15420572	Sel-moe-HKT4
	PACid_15412354	Sel-moe-HKT5
	PACid_15412619	Sel-moe-HKT6

At the functional level, the six HKTs of *S. moellendorffii* very likely share properties of the orthologs from other species. They may thus be implicated in K^+^/Na^+^ recirculation in this vascular plant and could contribute not only to K^+^ transport but in first line to desalination and Na^+^ detoxification.

### Voltage-independent K^+^ channels

**Figure 3:**
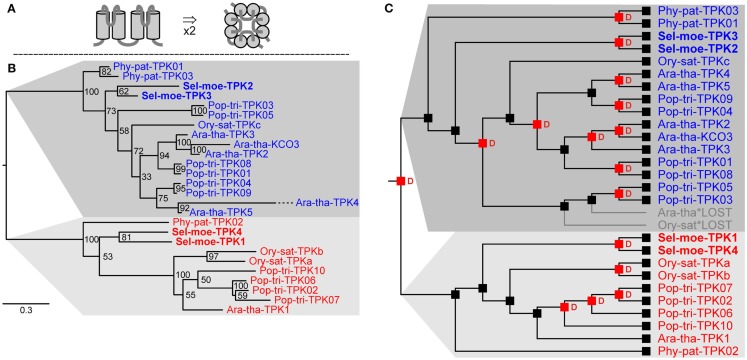
**Tandem-pore K^+^ (TPK) channels in land plants**. **(A)** Predicted structure of one subunit with a two-fold TM-P-TM motif in side view (left) and assembled functional channel dimer in top view (right). **(B)** Evolutionary relationships among TPK channels in land plants. There are two clear groups of TPK orthologs in extant land plants. **(C)** Reconciliation analysis of TPK channels. The last recent common ancestor of embryophytes had two genes coding for TPK channel subunits. Red “D”s at branching points indicate predicted gene duplications. Gray branches indicate gene losses.

Potassium channels play important roles in many physiological aspects of higher plants such as osmoregulation, turgor-driven movements, and ion uptake. It is estimated that K^+^ channels can contribute to more than 50% of the nutritional K^+^ uptake under most field conditions (Spalding et al., 1999; Amtmann and Blatt, 2009). In angiosperms there are two large groups of K^+^ channels: voltage-gated channels, the activity of which is regulated by the transmembrane voltage (Dreyer and Blatt, 2009), and non-voltage-gated K^+^ channels. Non-voltage-gated K^+^ channels form the class of tandem-pore K^+^ (TPK) channels. Functional TPK channels are proposed to form dimers consisting of two identical subunits (Maitrejean et al., 2011). Each subunit is characterized by a structure with four transmembrane domains and two pore-forming loops between the first and second and the third and forth membrane-spanning domain, respectively (Figure 3A; Voelker et al., 2010).

Searching for proteins with the characteristic pore-forming region, in the genome of *S. moellendorffii* four genes coding for TPK channel subunits could be identified (Table 3). Together with the six TPKs from *Arabidopsis* (Ara-tha-TPK1–5 and Ara-tha-KCO3), three from rice, ten from poplar, and three from *P. patens* they could be classed into two groups of orthologs (Figure 3B). This implies that the ancestor of land plants had already two of these genes. A deeper phylogenetic analysis revealed several duplication events in the two groups, both species-specific and at higher levels (Figure 3C). A remarkable example in this context is KCO3 from *A. thaliana*. This subunit lacks the first of the two pore loops and was originally considered as founder of a separate channel family with structural features (TM-P-TM; two transmembrane α-helices, and pore-forming P segment) similar to the simplest class of K^+^ channels from bacteria and animals. It became evident, however, that *Ara-tha-KCO3* developed through a very recent evolutionary event involving gene duplication of the *Ara-tha-TPK2* gene followed by partial deletion (Marcel et al., 2010; Voelker et al., 2010). And indeed, in line with this concept, neither the genome of *S. moellendorffii* nor that of *P. patens* appears to contain genes coding for K^+^ channels of the TM-P-TM type.

**Table 3:** **Two pore K^+^ (TPK) channels presented in this study**.

Species	Locus	Name
*A. thaliana*	AT5G55630	Ara-tha-TPK1
	AT5G46370	Ara-tha-TPK2
	AT4G18160	Ara-tha-TPK3
	AT1G02510	Ara-tha-TPK4
	AT4G01840	Ara-tha-TPK5
	AT5G46360	Ara-tha-KCO3
*O. sativa*	LOC_Os03g54100	Ory-sat-TPKa
	LOC_Os07g01810	Ory-sat-TPKb
	LOC_Os09g12790	Ory-sat-TPKc
*P. trichocarpa*	POPTR_0001s34510	Pop-tri-TPK01
	POPTR_0001s37550	Pop-tri-TPK02
	POPTR_0002s06010	Pop-tri-TPK03
	POPTR_0002s18870	Pop-tri-TPK04
	POPTR_0005s22460	Pop-tri-TPK05
	POPTR_0008s00520	Pop-tri-TPK06
	POPTR_0008s00530	Pop-tri-TPK07
	POPTR_0011s02810	Pop-tri-TPK08
	POPTR_0014s10900	Pop-tri-TPK09
	POPTR_0016s00890	Pop-tri-TPK10
*P. patens*	Pp1s114_5V6	Phy-pat-TPK01
	Pp1s334_27V6	Phy-pat-TPK02
	Pp1s9_180V6	Phy-pat-TPK03
*S. moellendorffii*	PACid_15414254	Sel-moe-TPK1
	PACid_15420903	Sel-moe-TPK2
	PACid_15415112	Sel-moe-TPK3
	PACid_15420585	Sel-moe-TPK4

TPK channels in plants were reported to be targeted to the vacuolar membrane (Czempinski et al., 2002; Voelker et al., 2006; Latz et al., 2007; Dunkel et al., 2008; Isayenkov et al., 2011a,b). The exception is Ara-tha-TPK4 which has been reported to be targeted also to the plasma membrane (Becker et al., 2004). However, orthologs of *Ara-tha-TPK4* were only found in the genus *Arabidopsis* so far (i.e., *A. thaliana* and *A. lyrata*; Voelker et al., 2010) but not in other plant species indicating a rather recent evolutionary event in channel specialization. Therefore, we have justified reasons to hypothesize that TPKs in bryophytes and lycophytes are vacuolar K^+^ channels. Their physiological role, however, remains as speculative as that of TPKs in other plants (Voelker et al., 2010).

### Voltage-gated K^+^ channels of the *Shaker*-type

**Figure 4:**
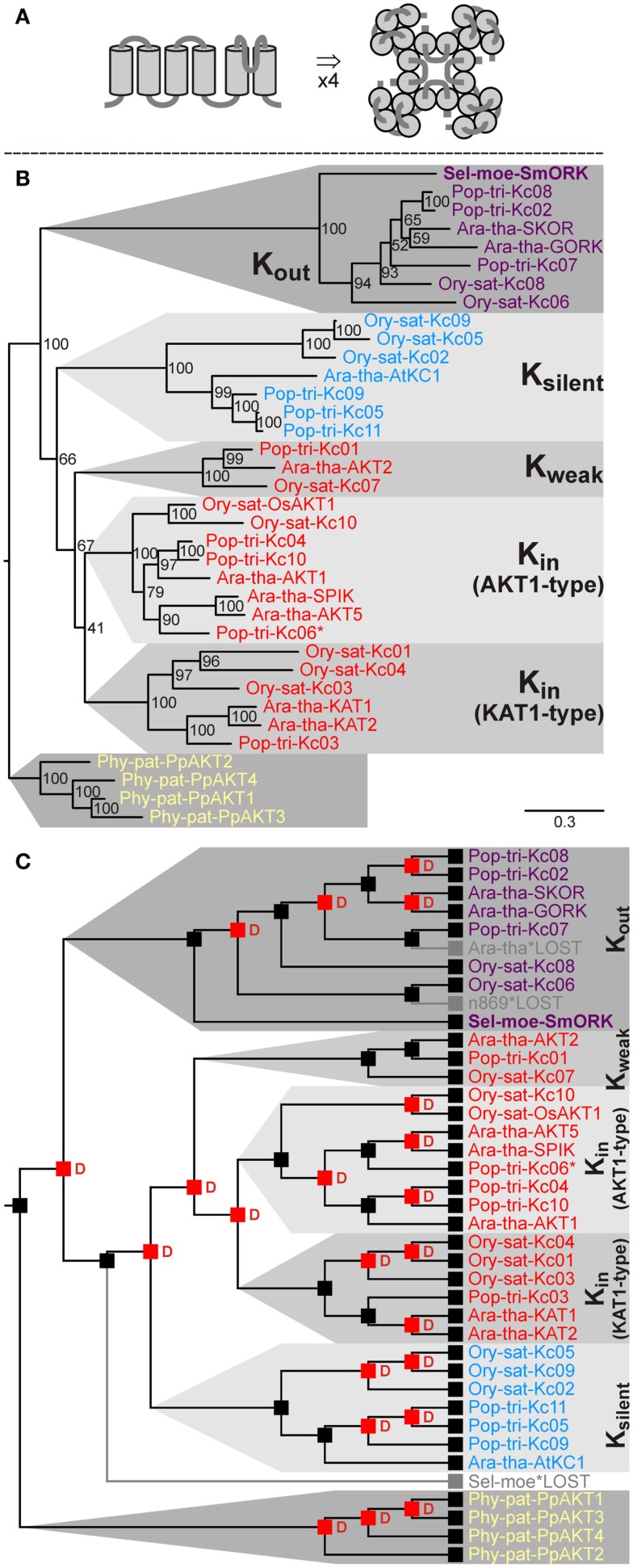
**Evolutionary relationships among voltage-gated *Shaker*-like K^+^ channels in land plants**. **(A)** Predicted structure of one subunit with a 5TM-P-TM motif in side view (left) and assembled functional channel tetramer in top view (right). **(B)** Evolutionary relationships among *Shaker*-like channels in land plants. Extensive functional analyses identified inward-rectifying (K_in_) channels, outward-rectifying (K_out_) channels, weakly rectifying (K_weak_) channels, and silent (K_silent_) channel subunits that assemble with K_in_ subunits and modulate K^+^ uptake channel properties. **(C)** Reconciliation analysis of *Shaker*-like K^+^ channels. The common ancestor of land plants had a single *Shaker*-like K^+^ channel. Since then several amplifications have occurred: a bryophyte specific amplification following the split between the lineage leading to *P. patens* and the tracheophytes, and duplications in the tracheophyte lineage. The common ancestor of tracheophytes had two genes coding for *Shaker*-like K^+^ channel subunits. One of these got lost in the lineage leading to *S. moellendorffii* after the split of angiosperms. Red “D”s at branching points indicate predicted gene duplications. Gray branches indicate gene losses. For Pop-tri-Kc06 only partial sequence information was available (indicated by an asterisk).

Voltage-gated K^+^ channels are tetrameric proteins built of four α-subunits. One subunit shows usually a structure with six transmembrane domains and one pore loop (5TM-P-TM). The first four transmembrane domains fold into the voltage-sensor module, and the pore loop together with the fifth and sixth transmembrane domains establishes the permeation pathway module (Figure 4A; Dreyer and Blatt, 2009).

Voltage-gated potassium channels in angiosperms are targeted to the plasma membrane and could normally be grouped into the class of *Shaker*-like K^+^ channels that subdivides into four functional subgroups: (a) Inward-rectifying (K_in_) channels open at membrane hyperpolarization and are responsible for K^+^ uptake. (b) Silent (K_silent_) channel subunits assemble with K_in_ subunits and modulate K^+^ uptake channel properties. (c) Weakly rectifying (K_weak_) channels are specialized K_in_ channels that show a bi-modal gating behavior. They appear to play a special role in the energy household of vascular tissues. (d) Outward-rectifying (K_out_) channel subunits open at depolarizing voltages and mediate K^+^ release, e.g., during xylem loading or stomata closure (see Dreyer and Uozumi, 2011, for a contemporary review). Our screening strategy based on the characteristic pore-forming region identified nine – already known – genes coding for *Shaker*-like channels in *Arabidopsis*, eleven in rice, and eleven in poplar. Genes coding for *Shaker*-like K^+^ channels were also identified in the moss *P. patens* and in the fern ally *S. moellendorffii* (Table 4). However, whereas four K_in_-like channels were identified in *P. patens*, despite a very careful screening strategy no such gene could be found in *S. moellendorffii*. Instead there, a gene coding for a K_out_ channel subunit was discovered as the only *Shaker*-like channel (Figure 4). This channel comprises all the essential structural features that were shown in the Ara-tha-SKOR K_out_ channel to be responsible for a unique K^+^ sensing property (Johansson et al., 2006). K_out_ channels open upon depolarization but additionally adjust their gating to the prevailing concentration of K^+^ outside. As a consequence, they open only at voltages positive of the K^+^ equilibrium voltage, *E*_K_, when the electrochemical driving force is directed outward and so ensure K^+^ efflux regardless of the extracellular K^+^ concentration. This ability to adapt channel gating to the cation concentration outside guarantees an efficient K^+^ release during xylem loading and stomatal closure, for instance, even under varying external K^+^ (from 10 nM to 100 mM; Blatt, 1988; Schroeder, 1988; Wegner and de Boer, 1997; Gaymard et al., 1998; Ache et al., 2000). From analogy we may postulate that the presence of a K_out_ channel in the vascular plant *S. moellendorffii* and its absence in the non-vascular plant *P. patens* is correlated with the important evolutionary step of vascularization. In contrast, it is rather difficult to find an explanation for the loss of the K_in_/K_weak_/K_silent_ channel branch in *S. moellendorffii*.

**Table 4:** **Voltage-gated *Shaker*-like K^+^ channels presented in this study**.

Species	Locus/protein ID	Name
*A. thaliana*	AT5G46240	Ara-tha-KAT1
	AT4G18290	Ara-tha-KAT2
	AT2G26650	Ara-tha-AKT1
	AT4G32500	Ara-tha-AKT5
	AT2G25600	Ara-tha-SPIK
	AT4G22200	Ara-tha-AKT2
	AT4G32650	Ara-tha-AtKC1
	AT3G02850	Ara-tha-SKOR
	AT5G37500	Ara-tha-GORK
*O. sativa*	LOC_Os01g45990	Ory-sat-OsAKT1
	LOC_Os01g11250	Ory-sat-Kc01
	LOC_Os01g52070	Ory-sat-Kc02
	LOC_Os01g55200	Ory-sat-Kc03
	LOC_Os02g14840	Ory-sat-Kc04
	LOC_Os04g02720	Ory-sat-Kc05
	LOC_Os04g36740	Ory-sat-Kc06
	LOC_Os05g35410	Ory-sat-Kc07
	LOC_Os06g14030	Ory-sat-Kc08
	LOC_Os06g14310	Ory-sat-Kc09
	LOC_Os07g07910	Ory-sat-Kc10
*P. trichocarpa*	POPTR_0003s01270	Pop-tri-Kc01
	POPTR_0004s08170	Pop-tri-Kc02
	POPTR_0004s13910	Pop-tri-Kc03
	POPTR_0006s15950	Pop-tri-Kc04
	POPTR_0006s26140	Pop-tri-Kc05
	POPTR_0006s26600	Pop-tri-Kc06
	POPTR_0012s04000	Pop-tri-Kc07
	POPTR_0017s02430	Pop-tri-Kc08
	POPTR_0018s00970	Pop-tri-Kc09
	POPTR_0018s06510	Pop-tri-Kc10
	POPTR_0242s00230	Pop-tri-Kc11
*P. patens*	Pp1s283_74V6	Phy-pat-AKT1
	Pp1s3_156V6	Phy-pat-AKT2
	Pp1s22_165V6	Phy-pat-AKT3
	Pp1s2_170V6	Phy-pat-AKT4
*S. moellendorffii*	453399	Sel-moe-SmORK

### Other types of voltage-gated K^+^ channels in algae, bryophytes, and lycophytes

In addition to *Shaker*-like channels the genomes of both, *S. moellendorffii* and *P. patens*, contain members of another class of putatively voltage-gated potassium channels (Table 5). These channels show some similarity with large conductance Ca^2+^-activated K^+^ channels (“big K” = BK channels), a channel type that is widely present in animals (including humans) but absent in flowering plants, for instance. BK channels activate in response to membrane depolarization and binding of intracellular Ca^2+^ and Mg^2+^ (Latorre et al., 2010). These channels are built of α- and β-subunits, where – as in *Shaker*-like channels – four α-subunits form the *per se* functional permeation pathway-establishing unit and the β-subunits just modulate and fine-tune channel properties. In contrast to *Shaker*-like channels the BK channel protein consists of seven (instead of six) transmembrane domains (6TM-P-TM structure) that lead to an exoplasmic N-terminus. Also BK-like channel α-subunits from *P. patens* and *S. moellendorffii* show a larger N-terminal region compared to plant *Shaker*-like channels. It might thus be speculated that also these proteins fold into a 6TM-P-TM structure instead of the 5TM-P-TM *Shaker*-like topology.

**Table 5:** **Voltage-gated K^+^ channels of other types presented in this study**.

Species	Locus/protein ID	Name
*A. thaliana*	–	–
*O. sativa*	–	–
*P. trichocarpa*	–	–
*P. patens*	XP_001753265	Phy-pat-BK1
	XP_001773545	Phy-pat-BK2
*S. moellendorffii*	PACid_15411641	Sel-moe-BK1
	PACid_15417632	Sel-moe-BK2
*C. reinhardtii*	Cre01.g022150.t1.1	Chl-rei-Kc01
	Cre07.g329882.t1.2	Chl-rei-Kc02
	Cre07.g330400.t1.2	Chl-rei-Kc03
	Cre10.g432550.t1.1	Chl-rei-Kc04
	Cre13.g594050.t1.1	Chl-rei-Kc05
	Cre13.g603750.t1.2	Chl-rei-Kc06
	Cre43.g787450.t1.1	Chl-rei-Kc07
	Cre02.g146300.t1.2	Chl-rei-Kc08
	Cre12.g531950.t1.2	Chl-rei-Kc09
	Cre02.g144950.t1.2	Chl-rei-Kc10
*Coccomyxa* sp.C-169	Genemark1.4196_g	Coccomy-Kc01
	Genemark1.7704_g	Coccomy-Kc02
	Genemark1.8069_g	Coccomy-Kc03
	estExt_fgenesh1_pg.C_190110	Coccomy-Kc04
*Micromonas* sp. RCC299	XP_002500200	Micromo-Kc01
	XP_002508929	Micromo-Kc02
	XP_002509136	Micromo-Kc03
	XP_002500877	Micromo-Kc04
	XP_002502332	Micromo-Kc05
	XP_002500929	Micromo-Kc06
	XM_002502171	Micromo-Kc07
	XM_002504550	Micromo-Kc08
	XM_002501933	Micromo-Kc09
*O. tauri*	Ot13g00490	Ost-tau-Kc01
	Ot11g00900	Ost-tau-Kc02
	Ot13g00630	Ost-tau-Kc03
	Ot01g04220	Ost-tau-Kc04
	Ot01g00450	Ost-tau-Kc05
*V. carteri*	PACid_17996094	Vol-car-Kc01
	PACid_18005696	Vol-car-Kc02
	PACid_17996282	Vol-car-Kc03
	PACid_18006137	Vol-car-Kc04
	PACid_18007561	Vol-car-Kc05
	PACid_18000814	Vol-car-Kc06
	PACid_18001906	Vol-car-Kc07
	PACid_18004030	Vol-car-Kc08
	PACid_18008362	Vol-car-Kc09

The functional properties and the physiological roles of plant BK-like channels are unknown. In mammalian tissues, BK channels serve as a negative-feedback mechanism for excitatory events that lead to increases in calcium concentration or membrane depolarization. In this way, they play a key role, for instance, in regulating the contractile tone in vascular smooth muscle cells or help to terminate the action potential and thus modulate secretion in chromaffin cells. It might be speculated that – at least in *S. moellendorffii* – the two BK-like channels could compensate for the absent *Shaker*-like K_in_ channels in carrying out functions in K^+^ uptake and distribution.

**Figure 5:**
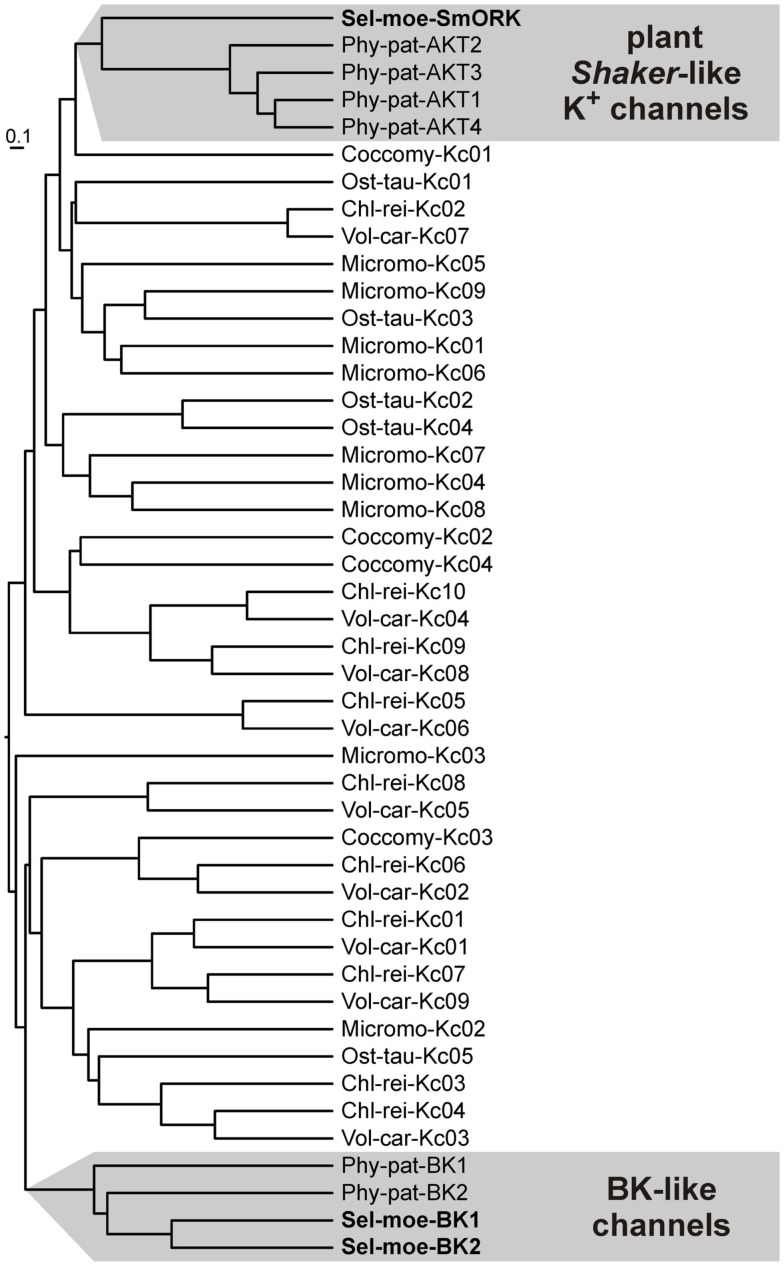
**Voltage-gated K^+^ channels in Chlorophyta, Bryophyta, and Lycophyta**. In contrast to land plants, voltage-gated K^+^ channels in algae show a large structural diversity. The functional variety of K^+^ channels in higher plants (Figure 4; *Shaker*-like K^+^ channels) developed from only one of these channel types.

To assess the evolutionary origin of the non-*Shaker*-like channels we screened the genomes of the green algae *Chlamydomonas reinhardtii*, *Coccomyxa* sp.C-169, *Micromonas* sp. RCC299, *Ostreococcus tauri*, and *Volvox carteri* for voltage-gated K^+^ channels. Despite the fact that this transporter class in algae exhibits a huge structural diversity comprising also homologs of plant *Shaker*-like channels (Figure 5), a clear trace leading to BK-like channels in *S. moellendorffii* or *P. patens* could not be identified. The two most similar channels from *Volvox* and *Chlamydomonas* share an identity of 16–19% over a stretch of ∼500 amino acids. In comparison, a BLAST search at NCBI^1^ limited to a query coverage of >45% resulted as best hit outside Animalia, *S. moellendorffii*, or *P. patens* in a voltage-gated K^+^ channel from *Phytophthora infestans* (XM_002998337) with ∼28% identity over a stretch of ∼500 amino acids. Unfortunately, from all these results we cannot resolve unequivocally the origin of BK-like channels in *S. moellendorffii* and *P. patens*. Neither can we exclude the possibility that bryophytes and lycophytes may have acquired these K^+^ channel genes from Fungi or Protozoa. However, our data (Figure 5) clearly indicate that the diversity of voltage-gated K^+^ channels observable in Chlorophyta collapsed contemporaneously with the transition of green plants from an aqueous to a dry environment. In higher plants the subsequent functional diversification into K_in_/K_out_/K_weak_/K_silent_ (Figure 4) took place on the basis of only one surviving channel class.

## Summary

In the haploid genome of the spike moss *S. moellendorffii* we identified 1 homolog of voltage-gated outward-rectifying K^+^ release channels, 4 homologs of voltage-independent tandem pore K^+^ channels, 2 homologs with some similarity to large conductance Ca^2+^-activated K^+^ (BK) channels, 11 homologs of transporters of the HAK type, as well as 6 homologs of the HKT type (Table 6). On the basis of phylogenetic analyses, detailed functional properties can be predicted for a few of them. Most probable is that Sel-moe-SmORK forms voltage-gated K^+^ release channels involved in stomatal closure and/or in K^+^ loading into the vascular bundles.

**Table 6:** **Summary – Molecular toolkit for K^+^ uptake and re-distribution in *S. moellendorffii***.

11	Genes coding for transporters of the HAK type
6	Genes coding for transporters of the HKT type
4	Genes coding for TPK subunits that dimerize into non-voltage-gated K^+^ channels
1	Gene coding for a subunit that homotetramerizes into voltage-gated K^+^ release (K_out_) channels
2	Genes coding for subunits that tetramerize into channels with some similarity to animal large conductance Ca^2+^-activated K^+^ (“big K” = BK) channels

## Materials and Methods

### Genome-wide search for K^+^ transporters

Putative K^+^ transporters were identified using the conceptual proteomes of *A. thaliana* (TAIR10 Genome release), *O. sativa spp. Indica*, *P. trichocarpa*, *P. patens*, and *S. moellendorffii* (Phytozome v6.0) and the algae genomes *Coccomyxa*_C169, *Micromonas* RCC299, *O. tauri* (v2, v3, and v4 respectively^2^), *V. carteri*, and *C. reinhardtii* (Phytozome v8.0) by screening with different transporter class-specific protein motifs: three motifs for K^+^ channels ((1) [S,T]-x-x-T-x-G-[Y,F,L]-G-[D,E], (2) R-[L,F]-x-R-[L,V,I,A,G]-x-[R,C,K]-[V,A,L,M], (3) [A,V,S]-Y-[L,I]-[I,L]-G-[N,I]-[M,I]-T-[N,A]-L-[V,I]); two motifs for HKTs ((4) [S,T,A]-x-[F,Y,V,L,C]-x-[D,N,S]-G, (5) [G,A]-[Y,F]-[G,A]-x-[V,A,I]-G-[L,M,Y,F]-[S,T]); and five motifs for HAK transporters ((6) [A,G]-[D,S,G]-[V,L,I,M]-x-x-[S,A]-P-L-Y; (7) [A,G]-[N,D,H,S]-[D,N]-x-G-[E,Q,D,N]-[A,G]; (8) [A,G,S]-[D,N]-[G,S,A,C]-x-[L,I,V,F]-x-P-x-[V,I,L,M]-[A,S]; (9) G-[S,A,T,C]-E-[A,G]-x-[F,Y]-A-[D,N,E]-[L,I,V]-[G,C,S,A]-x-F; (10) [Y,F]-x-x-x-x-x-[H,F,Y]-G-Y-x-[E,D]) using the FUZZNUC program from EMBOSS (Rice et al., 2000). Additionally, results were checked against BLAST searches in the five genomes using known transporters of different classes from *Arabidopsis* and rice as templates. In order to eliminate false-positives the resulting raw-data were curated in a semi-automatic way. In a first step sequences with a length <70% of the average length between the outermost motifs in the corresponding *Arabidopsis* transporters were discarded. Subsequently, the remaining *n* protein sequences of each transporter type of each species were pairwise aligned using ClustalW2^3^. From the resulting *n*(*n−*1)/2 pairs those with a score of <20 and of 100 (identical sequences) were removed. The residual pairs fragmented the sequences into distinct groups. That group with the highest similarity to the corresponding *Arabidopsis* transporters was selected for further analyses.

To verify whether the screening for K^+^ channels in *S. moellendorffii* was exhaustive, its genome was screened in the six-frame translations using the program SIXPACK from EMBOSS (Rice et al., 2000) for the presence of the K^+^-selectivity filter motif G-Y-G in ORFs. Following a positive hit, the closer environment of the GYG was inspected manually for further characteristic sequence features allowing categorizing the peptide to be part of a K^+^ channel. As a result – besides the K^+^ channels obtained already in the first screening – only the K_out_ channel SmORK could be identified in addition.

### Phylogenetic analyses

Sequences from each family were aligned using MAFFT (Katoh and Toh, 2010), and alignments were filtered using GBlocks (Castresana, 2000) in order to eliminate regions of low quality. Briefly, the minimum number of sequences for a conserved position was half the number of sequences, the minimum number of sequences for a flanking position was half the number of sequences, the maximum length of contiguous non-conserved positions was 20, and the minimum length of a block was two, positions with gaps were not treated differently from other position. Evolutionary relationships were inferred by Maximum Likelihood using RAxML and 1000 bootstrap replicates (Stamatakis, 2006). The evolutionary model used for phylogenetic analyses was inferred using ProtTest (Darriba et al., 2011). For two pore channels and HAK transporters the model was LG + γ, for HKTs and *Shaker*-like channels it was JTT + γ. In order to root and resolve the gene trees we performed a gene tree-species tree reconciliation analysis using the species tree from Lang et al. (2010; TreeBase 10409). Reconciliation analysis was carried out in Notung 2.6 (Chen et al., 2000; Vernot et al., 2007). To get an idea of the phylogenetic structure of the other voltage-gated K^+^ channels displayed in Figure 5, the sequences were hierarchically clustered based on pairwise identities between two sequences using UPGMA (Unweighted Pair Group Method with Arithmetic Mean). UPGMA analyses were carried out in MAFFT^4^.

## Conflict of Interest Statement

The authors declare that the research was conducted in the absence of any commercial or financial relationships that could be construed as a potential conflict of interest.

## Supplementary Material

The alignments used for generating the phylogenetic trees presented in this study are available online as Supplementary Material.

The Supplementary Material for this article can be found online at http://www.frontiersin.org/Plant_Evolution_and_Development/10.3389/fpls.2012.00167/abstract

## Footnotes

^1^http://blast.ncbi.nlm.nih.gov/Blast.cgi

^2^http://genome.jgi-psf.org/

^3^http://www.ebi.ac.uk/Tools/services/web/toolform.ebi?tool = clustalw2

^4^http://mafft.cbrc.jp/alignment/software/
